# Exploring physiotherapy students’ clinical experiences during educational disruption, through a community of practice lens

**DOI:** 10.4102/hsag.v31i0.3286

**Published:** 2026-10-02

**Authors:** Tammy-Lee Williams, Blake Boggenpoel, Danelle Hess, Tania Steyl, Shamila Gamiet, Vuyolwethu Madasa, Tak Wing Yu, Nondwe Mlenzana, Farhana Karachi, Joliana Phillips

**Affiliations:** 1 Department of Physiotherapy, Faculty of Community and Health Sciences, University of the Western Cape, Cape Town, South Africa; 2 Department of Interprofessional Education Unit, Faculty of Community and Health Sciences, University of the Western Cape, Cape Town, South Africa

**Keywords:** clinical readiness, community of practice, disruption, learning and teaching, physiotherapy education, student experiences

## Abstract

**Background:**

The COVID-19 pandemic profoundly disrupted health professions education, affecting final-year physiotherapy students at a pivotal stage in their transition to clinical practice.

**Aim:**

This study explores final-year physiotherapy students’ experiences amid heightened uncertainty.

**Setting:**

A historically disadvantaged university in South Africa.

**Methods:**

Guided by Wenger’s Communities of Practice (CoP) framework, this qualitative exploratory study examined how students navigated readiness, fear, support systems and perceived learning gaps. Semi-structured interviews were conducted with nine purposively selected final-year students and analysed thematically.

**Results:**

Participants (seven females, two males; mean age 22.6 ± 1.3 years) reported shared concerns regarding infection risk, the influence of personal protective equipment on clinical performance, degree completion, family safety and the impact of online learning on skill acquisition. However, support from the physiotherapy department reinforced their sense of belonging. Students identified shared resources, including WhatsApp groups, peer support, clinician mentorship and faculty access, as critical to reinforcing their practice.

**Conclusion:**

While shared pandemic-related concerns heavily influenced students’ readiness for clinical learning, their sense of belonging was reinforced by departmental support. Utilising a shared repertoire of communication and mentorship helped them successfully navigate these unprecedented challenges.

**Contribution:**

These findings highlight the importance of creating a supportive and adaptable learning environment during periods of disruption, which centres on clear communication and robust support mechanisms.

## Introduction

The coronavirus disease 2019 (COVID-19) pandemic brought about profound disruptions in health professions education, forcing academic institutions and clinical training sites to rapidly adapt to remote learning, limited patient interaction and enhanced infection control protocols (Rose 2020:2131–2132; Sandhu & De Wolf 2020:1764740). These disruptions significantly impacted final-year physiotherapy students, who were at a critical point in their transition from academic learning to clinical practice. Traditionally, this transition relies on structured clinical exposure, supervision and opportunities for collaborative learning, all of which were compromised by pandemic-related restrictions (Ferrel & Ryan 2020:e7492) Physiotherapy students, in particular, faced multifaceted challenges including fear of infection, anxiety about transmitting the virus to family members, uncertainty around clinical competence and concerns about meeting graduation requirements (Davies, Lawford & Chan 2024:e2067). Simultaneously, the shift to online learning platforms raised concerns about the adequacy of theoretical preparation, with students often reporting a loss of engagement and diminished confidence in their readiness for real-world clinical scenarios (Gardanova et al. 2023:1921; O’Doherty et al. 2018:130). These challenges not only disrupted the acquisition of clinical skills but also affected students’ professional identity formation, a key developmental milestone in health professions education (Rehman et al. 2024:130–140). Research suggests that professional identity evolves through participation in authentic, practice-based experiences and interactions with more experienced members of the profession, processes that were disrupted by social distancing and institutional lockdowns (Terrón-Pérez, Cortes-Amador & Portolés-Simeó 2024:349). To understand how students managed this complex transition during a period of crisis, this study is framed by Wenger’s (1999) *Communities of Practice* (CoP) theory. Communities of practice offers a valuable lens through which to interpret the learning experiences of healthcare students, particularly in contexts where social interaction, mentorship and shared practices are essential. A community of practice is defined by a shared domain of interest (for example, physiotherapy), mutual engagement (for example, collaboration among students, lecturers and clinicians) and a shared repertoire of practices (for example, clinical skills, norms and values) (Wenger 1999). Within this framework, learning is not merely the acquisition of knowledge but a process of becoming, moving from peripheral to full participation in a professional community (Wenger 1999). Existing literature applying CoP in health professions education has shown its relevance in fostering clinical competence, collaborative learning and professional socialisation (Figurska 2012; Li et al. 2009). However, few studies have examined how these dynamics play out under crisis conditions such as a pandemic, where normal patterns of participation are disrupted. This study aims to explore the lived experiences of final-year physiotherapy students entering clinical placements during times of uncertainty and disruption, with a focus on how they navigated readiness, fear, support systems and perceived gaps in their learning. By framing the findings within the CoP model, this study offers insight into how social learning processes were maintained, adapted or fractured in the context of extreme disruption, and how educational communities can better support students through future crises.

## Research methods and design

This study was grounded in an interpretivist paradigm and adopted a qualitative exploratory design. Exploratory designs are particularly appropriate for research aiming to gain new insights into under-researched phenomena, such as students’ clinical education experiences during a global health crisis (Hunter, Mccallum & Howes 2019:1–7). A purposive sampling strategy was used. The authors were physiotherapy lecturers seeking answers during a pandemic, and the students were purposely recruited, based on their discipline as well as their year level of study, as only fourth-year students were returning to clinical placement during the pandemic. The researchers were lecturers who were actively involved in the education of the students who participated in the study. The researchers, therefore, acknowledged the possible power dynamics at play and noted that this factor could influence the students’ participation and how they answered the questions. The researchers attempted to mitigate this by dividing the data collection among at least three lecturers as well as providing students with the information sheet detailing the benefits and risks of participation in the study. The CoP framework, as outlined above, was used to guide the write-up of the results and discussion sections.

### Study participants

The study included nine final-year physiotherapy students: seven female and two male participants, with a mean age of 22.6 years (s.d. ± 1.3). Participants were recruited by the authors, who were also lecturers in the department. To address the potential power dynamics inherent in lecturer-student relationships, several ethical and methodological precautions were taken. These included dividing the data collection among three lecturers, providing clear information sheets outlining the voluntary nature of participation, and ensuring that participants were aware that their decision to participate (or not) would not affect their academic standing.

### Data collection

Data were collected through semi-structured, individual interviews by three members of the research team (DH, SG, JP). It allowed for both guided questioning and the flexibility to probe further based on participants’ responses. All interviews were conducted virtually via Google Meet due to COVID-19 safety protocols. Each interview lasted approximately 20 min – 30 min and was audio-recorded with participant consent. The interview guide is available in Table 1.

**TABLE 1:** Semi-structured interview guide.

Interview guide questions:	Categories
What are you most concerned about (going into clinical practice)?	Preparedness Risks Personally Family
What messaging and behaviours do you need from the department as you return to clinical practice?	Preparation Information Support
What are the tangible sources of support you believe would be most helpful as you return to clinical practice?	Department Supervisors Clinicians

Data saturation was reached following nine interviews; therefore, no new insights or themes emerged (Austin & Sutton 2014:436–440).

### Data analysis

The interviews were transcribed professionally. The authors analysed the data using the six-step thematic analysis method by Braun and Clarke (2021:1–100). The authors familiarised themselves with the data by reading the transcripts. Interesting and repeating insights were coded, and the data were organised according to the created codes, followed by the organisation of codes into themes. Thereafter, each member of the research team conducted steps one to four independently, and the team then met to review the themes. Finally, a manuscript was created which included extracts from the participants’ views. Validity and reliability of the data were ensured through various methods. Member checking was conducted by sending the transcripts to a few participants for validation. Triangulation within the research team (including those involved in data collection and others who were not) provided an external lens to minimise bias. One of the authors involved in the data collection or interviews also assisted with the personal protective equipment (PPE) training. All the members of the research team were lecturers in the department, which introduced some bias but also allowed for contextual insight into the students’ learning environment. This dynamic could allow assumptions to steer the data interpretation; however, to address reflexivity, all members of the research team were given a copy of the transcripts and audio recordings, independently coded the transcripts, met on more than one occasion to collaboratively discuss codes and themes, challenged interpretations and questioned emerging themes. This iterative dialogue ensured that the codes and themes created were a true reflection of the transcripts. This collaborative, multi-perspective approach enhanced the credibility and confirmability of the analysis as the roles of researcher and lecturer were confronted.

### Ethical considerations

Ethical clearance to conduct this study was obtained from the University of the Western Cape Humanities and Social Science Research Ethics Committee (No. HS20/6/4).

## Results

The findings are best understood through the lens of Wenger’s CoP (1999), which emphasises learning as a social process shaped by participation, shared goals and mutual engagement. As final-year physiotherapy students transitioned to clinical placements during a time of uncertainty, their experiences reflected both a disruption in traditional learning practices and a strong reliance on existing and emerging communities for support and development. In the Domain category, four sub-themes emerged, namely clinical performance, Inability to complete degree, Family safety and Influence of mode of learning on knowledge and skill acquisition. In the Community category, the sub-themes identified included student advocacy and responsive departmental support as well as ongoing communication and emotional support. In the Practice category, the sub-themes identified included Peer support, Clinician support and immediate learning support. Three main and eight sub-themes emerged from the data analysis. Figure 1 represents these themes and sub-themes.

**FIGURE 1:**
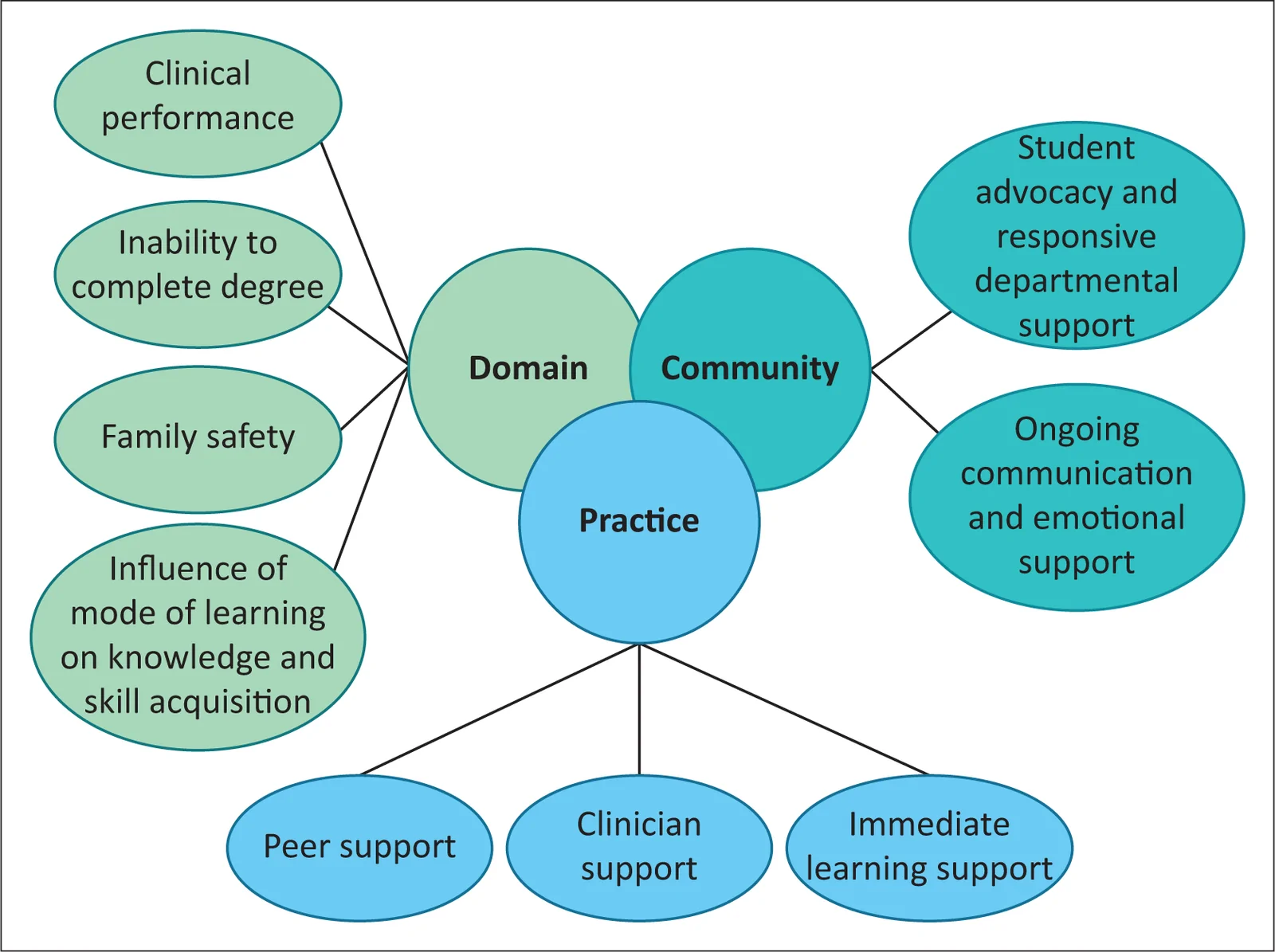
Themes and sub-themes according to the community of practice framework.

### Theme: Domain

#### Sub-theme: Clinical performance

Concerns about clinical performance reflect the *Domain dimension* of Wenger’s (1999) CoP framework as a shared domain of interest among the students, influencing their professional identity. The dual demands of health and safety combined with clinical expectations created an experience of cognitive and emotional overload for the students, impacting their ability to focus on learning opportunities during the clinical platform. The added burden of PPE and safety protocols disrupted students’ ability to fully participate in these practices, challenging their development of competence, communication and confidence as emerging members of the physiotherapy community. The following is an example of a student’s report:

‘I think also, a big, big concern of mine is are we going to have the same level of care that we’ve had previously, in terms of, having that same connection, maybe, or rapport with the patient, because now you’ve got a mask, you’ve got a visor, … now you’ve got to try … being hands-on, but also being safe.’ (Participant 6, 4th Year Female, Physiotherapy Student)

#### Sub-theme: Inability to complete degree

The shared fear of not completing clinical hours and fulfilling the requirements belonging to the profession relates to the *Domain dimension* of Wenger’s (1999) CoP framework. Students reported feeling fearful of contracting the COVID-19 virus during their clinical training and the inability to complete the clinical hours required for the attainment of their physiotherapy degree. The following is an example of a student’s report:

‘I am scared that I might get COVID-19 within my block, and then I’d have to quarantine for fourteen days, and then after that, then I have fourteen days’ worth of, like, clinical practice hours that I haven’t done. So, I’ll be a bit behind, so that is my biggest fear.’ (Participant 3, 4th Year Female, Physiotherapy Student)

‘I’m concerned about actually getting corona, because … that is my most concern at this point, and actually … not actually being able to finish a block because of corona. Like, because there’s 14 days isolation, whereby you have to sit back and actually isolate yourself and stuff like that. That is my number one concern, because I’m not even sure if it means that I will not be able to finish the whole year, you see?’ (Participant 1, 4th Year Female, Physiotherapy Student)

#### Sub-theme: Family safety

The students’ concern for transmitting COVID-19 to their families reflects Wenger’s (1999) *Domain dimension* of the CoP framework as a shared concern which influenced their sense of belonging, responsibility and readiness to engage in clinical practice. This is illustrated by participant 4:

‘I also don’t want to bring anything back to my parents or my family, or anything, because I wouldn’t want to put them at risk in any sort of way. So, I think that my main concern at the moment is just keeping everybody around me safe.’ (Participant 4, 4th Year Male, Physiotherapy Student)

#### Sub-theme: Influence of mode of learning on knowledge and skill acquisition

The challenges students faced with the shift to online learning also reflect the *Domain dimension* of Wenger’s (1999) CoP framework. This shared concern left some students feeling underprepared for the clinical domain, particularly in high-stakes settings like the intensive care unit (ICU). This was evident in the students’ concerns, as stated below:

‘I have to be honest with that one, because I would say that I feel a little bit rough when it comes to my work, because I don’t feel like I am ready. I feel like most of my theory work that I’ve learnt, right now this year, was online, so even with that, for me personally … I don’t feel like I had enough information this year, as compared to the previous year, because the previous years we had class interactions.’ (Participant 1, 4th Year Female, Physiotherapy Student)

This theme was not unexpected, as undergraduate students have historically experienced difficulties engaging with online learning modalities within clinically-oriented disciplines such as Physiotherapy (Arsovski & Chichevska-Jovanova 2025:33–41). The findings demonstrate that the pandemic shifted the students’ shared *Domain* from one of acquiring clinical skill, to pressures of health safety and fragmented education threatening their progression into the profession.

### Theme: Community

#### Sub-theme: Student advocacy and responsive departmental support

Participants expressed a need for an empathetic and flexible department that acknowledged their concerns, respected individual circumstances and supported their decisions during challenging situations. The appreciation students expressed for lecturer support reflects the *Community dimension* of Wenger’s (1999) CoP framework. Through reassurance and advocacy, lecturers fostered a sense of belonging within the professional community, reducing anxiety and supporting students’ continued engagement in learning and identity formation despite the challenges of the pandemic. The quote below is an example of a participants’ experience:

‘… the Department can just be a bit more lenient with regards to our clinical performance, and just be a bit more understanding than usual.’ (Participant 5, 4th Year Female, Physiotherapy Student)

‘I think it’s a very nice idea that concern is shown from a lecturer, because, you know, sometimes people think they don’t worry about us and how we feel, and how we deal with stress. … [*name given*] was always communicating with us in the beginning and telling us: guys, you know what, I understand your stress … and it’s nice to get that kind of support, you know.’ (Participant 2, 4th Year Male, Physiotherapy Student)

#### Sub-theme: Ongoing communication and emotional support

Participants valued regular communication from the department and lecturers during clinical placements. Ongoing contact, emotional reassurance and opportunities for check-ins helped them feel supported, reduced feelings of isolation and uncertainty and strengthened their confidence and sense of belonging. Within Wenger’s (1999) *Community domain*, these interactions reflect mutual engagement by fostering connectedness and supportive relationships within the community of practice:

‘I feel like the Department did put that into place already by providing a support system, the WhatsApp group that we can just ask questions if we need to ask anything, if we need any help with anything.’ (Participant 5, 4th Year Female, Physiotherapy Student)

‘I think it’s a very nice idea that concern is shown from a lecturer, because, you know, sometimes people think: ag, he’s just going to give us work now, they don’t worry about us, and how we feel, and how we deal with stress. And, I think that many people like to think that [*name redacted – the head of department*] was always communicating with us in the beginning and telling us: guys, you know what, I understand your stress and you do this … and it’s nice to get that kind of support, you know. And even a random message from you [*as the clinical coordinator*] is also nice. That, okay, you know, people are thinking about us, that we’re in their prayers and things, so that also boost our morale.’ (Participant 2, 4th Year Male, Physiotherapy Student)

During a period of disruption to learning and teaching, the students’ professional *Community* is sustained through relational care, where active departmental support and consistent communication prevent student isolation and preserve their sense of belonging.

### Theme: Practice

#### Sub-theme: Peer support

The students’ recommended peer support, which reflects the *Practice dimension* of Wenger’s (1999) CoP framework as a shared repertoire to support and improve their practice, through sharing coping mechanisms, shared clinical reasoning or problem-solving collectively. Peer support was thus seen as an integral part in assisting students with problem-solving strategies related to specific clinical scenarios through shared learning experiences. These shared learning experiences thus create opportunities for improved clinical reasoning and confidence among students on their clinical placements. In addition, strategies such as these also minimise the effects of isolation, thus improving mental well-being and allowing students to engage in clinical learning:

The quote below displays the students’ feelings:

‘… because we’re not going to campus on Fridays, so we don’t have that interaction time with our lecturers and our peers. So, I think having a support group would be a good idea ….’ (Participant 7, 4th Year Female, Physiotherapy Student)

‘Or just checking in on people … if maybe there are students who are … suffering from anxiety … just to make sure everybody is still staying afloat, and that you know, can finish their [*clinical*]block.’ (Participant 2, 4th Year Male, Physiotherapy Student)

Peer learning and support are key mechanisms for enhancing learning outcomes and facilitating coping among peers. This sub-theme underscored the need for researchers and educators to intentionally cultivate opportunities for peer learning and support, not only in response to disruptions to learning and teaching, but also as an integral component of routine clinical teaching and training.

#### Sub-theme: Clinician support

The students also recommended greater clinician support while on the clinical practice platform, which aligns with the *Practice domain* of Wenger’s (1999) CoP framework to further support their practice. By seeking closer supervision and mentorship, students emphasised the importance of clinicians in scaffolding their learning and modelling professional practice. The students expressed a wish for more monitoring while they were assessing and treating patients. This served as an opportunity for clinicians to bridge the gap between theory and practice. Thereby inadvertently boosting students’ self-confidence and clinical competence in relation to patient management:

‘I think the most important one is actually getting support from clinicians, especially because I think they’ve worked during this time, right, and they have more experience during this time.’ (Participant 1, 4th Year Female, Physiotherapy Student)

#### Sub-theme: Immediate learning support

The need for immediate learning support reported by the students reflects Wenger’s (1999) *Practice domain*, emphasising shared repertoire and mutual engagement. Students expressed their need for immediate learning support from the departmental lecturers and clinical supervisors when their clinician at the clinical placement was not available. By requesting access to lecturers and supervisors through WhatsApp groups or video calls, students highlighted the value of timely collaboration and knowledge sharing, which would directly influence the development of their competence in their practice, as students could receive guidance on clinical decisions in real time. The quote below displays the students’ feelings:

‘WhatsApp group that we can just ask questions if we need to ask anything, if we need any help with anything.’ (Participant 5, 4th Year Female, Physiotherapy Student)

‘The most important one is actually getting support from clinicians, especially because I think they’ve worked during this time, right, and they have more experience during this time. So, I would love to also for them to actually tell us what stuff that we should look out for, I think for each and every placement there’s different ways of actually handling this pandemic.’ (Participant 1, 4th Year Female, Physiotherapy Student)

The sub-theme, ‘immediate learning support’ highlighted the need for prompt response when seeking clarification regarding clinical knowledge. The continuous availability of lecturers and supervisors on the WhatsApp platform offered valuable support, which helped put participants at ease within their clinical environments. During times of educational disruption, when undergraduate students experience significantly heightened anxiety, immediate learning support transitions from optional to essential, compelling lecturers and supervisors to prioritise their needs. Ultimately, the *Practice* theme demonstrates that peer collaboration, clinician mentorship and real-time supervision function as essential mechanisms that allow students to bridge the gap between theory and practice.

## Discussion

This study explored the experiences of final-year physiotherapy students entering clinical placements during times of uncertainty and disruption. Using Wenger’s (1999) CoP as a theoretical lens, the findings illustrate how students navigated complex emotional, educational and professional challenges by relying on the support of peers, educators and clinicians. The CoP framework is particularly useful for understanding the students’ desire for connection, mentorship and progression within the professional physiotherapy community during a time of unprecedented disruption (Li et al. 2009; Roberts 2006).

### Communities of practice: Domain

#### Clinical performance

Through the CoP lens, the key issues which were reported by participants were classified under the *Domain* dimension. These issues included concerns regarding the burden of PPE and safety protocols, which were perceived to impact their clinical performance and the development of competence as emerging members of the physiotherapy community (Stoffels et al. 2022:1423–1441).

#### Inability to complete degree and family safety

Furthermore, within the *Domain* dimension, students commonly expressed fear of contracting COVID-19 during clinical training, which they felt could affect their ability to complete their degree and risk spreading the virus to vulnerable family members. These findings coincide with Cantero-Garlito et al. (2021:1621), who reported that 34.6% of their study participants feared contracting the virus, 49.7% were concerned about infecting their families, and 47.6% were concerned about how the pandemic may affect their studies. Various authors have reported similar concerns and psychological distress among occupational therapy, physiotherapy students and medical students (Alshehri et al. 2023:411; Cao et al. 2020:112934; Tan 2024:38–58). Apart from the COVID-19 pandemic, Greeff et al. (2021:78–103) reported that first-year students experienced psychological distress during the Fees Must Fall protests, as they were faced with increased academic pressure due to missed classes and thoughts of dropping out or extending the length of their course. These findings highlight the similarities in concerns and psychological distress among health professional students during any disruption to teaching and learning.

#### Influence of mode of learning on knowledge and skill acquisition

Another key issue found among the students, classified under the *Domain* dimension of the CoP, was the influence of the mode of learning on their knowledge and skill acquisition. Students in this study reported that the transition to online learning reduced interaction with both peers and lecturers, which negatively influenced their knowledge acquisition. Similarly, Phadke, Hakim and Tilak (2021:6683–6687) found that in-person learning was more rewarding due to human interaction. In contrast, Tang et al. (2023:100) reported no impact of online learning on university students’ knowledge acquisition. The discrepancy may be attributed to differences in year levels, as the aforementioned studies investigated first-year students’ knowledge of basic fundamental concepts, whereas the present study focused on final-year students’ understanding of more complex concepts and conditions. Furthermore, Phadke et al. (2021:6683–6687) highlighted that the lack of human interaction, which is usually accompanied by the online environment, may create some degree of isolation for students. This was emphasised by one of the students in the current study who stated that they were required to work independently. Goudarzi, Onozawa and Takahashi (2024:66) found that online and hybrid educational approaches during the COVID-19 pandemic were equally effective in comparison to in-person instruction. This finding suggests that students in the current study preferred traditional teaching practices and found the transition from in-person to online learning challenging. It highlights the need for educational strategies that can bridge the gap between online theoretical learning and practical clinical preparedness. As advocated by Gardanova et al. (2023:1921), health professions education should adopt a forward-thinking approach which includes developing flexible curricula that are able to adapt to sudden disruptions and investing in faculty development to enhance preparedness for various teaching and learning scenarios. Goudarzi et al. (2024:66) further highlight that well-designed online and hybrid courses can enhance various aspects of learning, not only during pandemics, but as viable alternatives for future educational disruptions.

### Communities of practice: Community

#### Student advocacy and responsive departmental support

In the *Community* dimension of the CoP framework, the students expressed appreciation for the support provided by the Physiotherapy Department, which strengthened the students’ sense of belonging within the professional community. The Physiotherapy Department’s efforts to deliver up-to-date information and mandatory online training further contributed to students feeling prepared to continue their clinical training during the pandemic. The findings of this study contrast with Canet-Vélez et al. (2021:103072), where medical students reported lacking practical instruction regarding PPE. The students in this study received face-to-face PPE training from a healthcare professional specialising in infection prevention and control, and they practised donning and doffing in class. In-person PPE training was found to be the superior method to ensure compliance with the correct procedure of donning and doffing (Li et al. 2009:11).

#### Ongoing communication and emotional support

Our study reinforces the findings of Greeff et al. (2021:78–103) regarding the need for increased communication and transparency, particularly during crises and disruptions to usual academic activities. Institutions may consider establishing clear, multi-channel communication strategies, regularly updating and disseminating information from reliable sources and providing platforms for students to voice their concerns and receive timely responses. In this study, students felt supported by lecturers who adopted open communication and conveyed regular messages of motivation. In a review by Billings et al. (2021:1–17), the authors demonstrated that students showed appreciation for leaders who were accessible and visible throughout a crisis while delivering well-coordinated communication.

### Communities of practice: Practice

#### Peer support and clinician support

The sub-themes emerging in the *Practice* dimension of the CoP framework consisted of recommendations for peer support, clinician support and immediate learning support as resources which would support their practice. Support in the clinical environment has a strong influence on a positive learning experience (O’Doherty et al. 2018:130). Furthermore, the relationships between students, clinicians and supervisors at the clinical placement also positively influenced students’ development of self-confidence and competence (O’Doherty et al. 2018:130). According to Markowski et al. (2021:1519–1541), effective learning is fostered through engagement with peers and more experienced practitioners, promoting full participation in the healthcare teams’ community of practice. This supports the findings of the present study that peer and clinician support serve as important resources for reinforcing students’ practice. These findings highlight the importance of a comprehensive support system when preparing students for clinical practice.

### Strengths and limitations of the study

This study provides an in-depth understanding of final-year physiotherapy students’ experiences during a period of significant educational disruption, using Wenger’s CoP framework to generate theoretically grounded insights. The qualitative design allowed for rich exploration of students’ perceptions; however, the findings are limited to a single institution and may not be transferable to all physiotherapy programmes or healthcare disciplines. As the study explored experiences during the COVID-19 pandemic, some findings may be context-specific, although they offer broader insights into managing future disruptions.

### Recommendations

The overall finding of this study offers recommendations for physiotherapy departments preparing for the next disruption in clinical education. Structured and regular communication proved to be an important aspect in minimising uncertainties and strengthening students’ sense of belonging in the professional community. Firstly, the communication pathways should be formalised, including regular meetings, rapid information sharing and avenues for students to access support. Secondly, the results underscore the importance of anticipatory psychological and emotional care during disruptions. The inclusion of student well-being strategies, peer support networks and opportunities to reflect could help students manage their anxiety and remain engaged in their clinical learning. Thirdly, students appreciated having access to lecturers, clinical educators and other students in a timely manner when problems arose in practice. Future preparedness plans should thus contain provision of immediate academic and clinical support, including opportunities for virtual consultation, virtual CoP, and digital communication platforms. Finally, the lack of preparedness during online education indicates that departments should invest in flexible curricula and incorporate simulation and hybrid education methods, as well as contingency plans that ensure the continued development of clinical competence when in-person placements are affected. Together, they can contribute to building the educational system’s resilience and sustaining learning in the event of a future pandemic, disruption of the institution, or unexpected events. Future research should examine whether the support strategies identified in this study improve students’ preparedness, resilience and clinical competence across different institutions and health professions.

## Conclusion

Participants expressed shared concerns regarding competence, professional identity and collective responsibility for the safety of others, which influenced their readiness to participate in the domain of clinical learning during a period of uncertainty and disruption to traditional learning and teaching practices. The students’ sense of belonging within the community was reinforced by the support provided by the Physiotherapy Department during this time (such as communication, emotional support, adaptable learning pathways and clinical mentorship). Lastly, the shared repertoire, recommended through various support structures, served to reinforce the students’ practice amid these uncertain times. These findings highlight the importance of creating supportive, adaptable learning environments that strengthen students’ resilience, sense of belonging and professional identity during periods of disruption. Preparing institutions with flexible educational strategies, clear communication pathways and robust support mechanisms may enhance students’ readiness for clinical learning and help sustain professional development during future disruptions to healthcare education.
